# Not the age but education and vitamins a secret to good auditory memory and attention

**DOI:** 10.1002/alz.093592

**Published:** 2025-01-03

**Authors:** Deepashri Agrawal, Meenakshi Menon, Rajitha Narayanasamy, Palash K Malo, Abhishek Mensegere Lingegodwa, Meghana R, Vindhya Vishwanath, Divya N Mallikarjun, Amitha C M, Goutham Velavarajan, Ajith Partha, Prathima Arvind, Shafeeq K Shahul Hameed, Sunitha HS, Sadhana Singh, Albert Stezin, Jonas S. Sundarakumar, Thomas Gregor Issac

**Affiliations:** ^1^ Centre for Brain Research, Indian Institute of Science, Bangalore, Karnataka India

## Abstract

**Background:**

Auditory attention and memory are the understudied aspects of cognition. Poor performance on cognitive tasks is assumed to be due to peripheral hearing loss, which is not always the case. Auditory processing issues may affect the auditory recall and attention tasks even though the hearing and cognition are normal. Education and nutrition influence cognitive function, including auditory attention and memory. Education provides individuals with strategies essential for effective auditory processing, while nutrition plays a crucial role in supporting brain health and optimal cognitive functioning. This study aimed to investigate the relationship between education, nutrition, and auditory cognition in adults, especially when the peripheral hearing and CDR scores are normal.

**Method:**

Educational backgrounds were assessed based on years of schooling and academic achievements, nutritional status was evaluated through blood sample biochemistry. The hearing is assessed through a handheld audiometer, and cognition through a CDR score. Auditory attention and memory through standardized cognitive tests, immediate and delayed recall and Digit span forward and backwards.

**Result:**

The results revealed that education played a very significant role in our study. Subject with postgraduate and above education levels performed better compared to primary education level on auditory attention (B(S.E.) = ‐0.49 (0.19), p < 0.01), Logical memory‐ immediate (B(S.E) = ‐3.93 (0.85), p<0.001) and delayed recall (B (S.E) = ‐4.5 (0.86), p<0.001) tasks and Digit Span forward (‐1.2 (0.35), p<0.01) and digit span backward tasks (B (S.E) = ‐0.98 (0.27), p<0.001). Additionally, older individuals with Vitamin D insufficiency (B (S.E.) = ‐0.44 (0.2), p < 0.05) and Vitamin B12 deficiency exhibited poorer auditory attention, Immediate memory (B (S.E.) = ‐7.2 (2.90), p < 0.05) and delayed memory (B (S.E.) = ‐6.3 (2.91), p < 0.05) performance, respectively.

**Conclusion:**

This study highlights the role of auditory processing and importance of good education and proper nutrition in supporting auditory cognition. Investing in quality education and promoting healthy dietary habits, including adequate intake of Vitamin D and Vitamin B12, can contribute to better outcomes, particularly in the domain of auditory cognition. These findings underscore the need for proper hearing assessments, diet, and education for comprehensive interventions to optimize cognitive functions.

